# SARS-CoV-2: preliminary study of infected human nasopharyngeal tissue by high resolution microscopy

**DOI:** 10.1186/s12985-021-01620-1

**Published:** 2021-07-18

**Authors:** Brian Mondeja, Odalys Valdes, Sonia Resik, Ananayla Vizcaino, Emilio Acosta, Adelmo Montalván, Amira Paez, Mayra Mune, Roberto Rodríguez, Juan Valdés, Guelsys Gonzalez, Daisy Sanchez, Viviana Falcón, Yorexis González, Vivian Kourí, The IPK Virology Research Group, Angelina Díaz, María Guzmán

**Affiliations:** 1 Center for Advanced Studies of Cuba (CEA), La Habana, Cuba; 2 Institute of Tropical Medicine “Pedro Kourí” (IPK), La Habana, Cuba; 3 Institute of Cybernetics, Mathematics, and Physics of Cuba (ICIMAF), La Habana, Cuba; 4 Center of Genetic Engineer and Biotechnology of Cuba (CIGB), La Habana, Cuba

**Keywords:** Coronavirus, SARS-CoV-2, SEM, AFM, Microscopy

## Abstract

**Background:**

The novel coronavirus SARS-CoV-2 is the etiological agent of COVID-19. This virus has become one of the most dangerous in recent times with a very high rate of transmission. At present, several publications show the typical crown-shape of the novel coronavirus grown in cell cultures. However, an integral ultramicroscopy study done directly from clinical specimens has not been published.

**Methods:**

Nasopharyngeal swabs were collected from 12 Cuban individuals, six asymptomatic and RT-PCR negative (negative control) and six others from a COVID-19 symptomatic and RT-PCR positive for SARS CoV-2. Samples were treated with an aldehyde solution and processed by scanning electron microscopy (SEM), confocal microscopy (CM) and, atomic force microscopy. Improvement and segmentation of coronavirus images were performed by a novel mathematical image enhancement algorithm.

**Results:**

The images of the negative control sample showed the characteristic healthy microvilli morphology at the apical region of the nasal epithelial cells. As expected, they do not display virus-like structures. The images of the positive sample showed characteristic coronavirus-like particles and evident destruction of microvilli. In some regions, virions budding through the cell membrane were observed. Microvilli destruction could explain the anosmia reported by some patients. Virus-particles emerging from the cell-surface with a variable size ranging from 80 to 400 nm were observed by SEM. Viral antigen was identified in the apical cells zone by CM.

**Conclusions:**

The integral microscopy study showed that SARS-CoV-2 has a similar image to SARS-CoV. The application of several high-resolution microscopy techniques to nasopharyngeal samples awaits future use.

**Supplementary Information:**

The online version contains supplementary material available at 10.1186/s12985-021-01620-1.

## Background

The family Coronaviruses comprises a wide-ranging of viruses that infect many animal species including human beings[1]. In December 2019, a new coronavirus disease emerged in China. Rapidly, the disease caused by the SARS CoV-2 extended to the whole world with a record in the number of cases and fatalities [2–5]. SARS-CoV-2 is 96.2% identical to a bat coronavirus at the whole-genome level and it belongs to the species of SARS-CoV [6]. Due to the genetic similarity to SARS-CoV, several characteristics have been assumed, but not clarified. Therefore, more deep investigations with a precise specific morphological description of this novel virus are necessary [5, 7].

SARS-CoVs are enveloped viruses with a diameter ranging from 60 to 400 nm. The envelope contains the envelope (E) and spike (S) proteins. The viral RNA is surrounded by the nucleocapsid. The overall structure looks similar to other viruses of the *Coronaviridae* family. The S-protein forms a clover-shaped trimer, with three S1 heads and a trimeric S2 stalk. The envelope-anchored spike protein guides coronavirus entry into host cells. This state of the spike on the mature virions is called “prefusion” [1, 8–10]. After viral replication and before the viral release, the spike proteins move from the Golgi apparatus to cell membrane, and the ribonucleoprotein core interact with this S-rich membrane. Not microscopic evidence of this final stage has previously been shown for SARS-CoV-2, but following its similarity with SARS-CoV, understanding its historical similarity with that of this characteristic has been assumed [8].

The structural details of the SARS-CoV-2 virus are essential towards understanding its historical resembles that of other coronaviruses (mode of infection, mechanism of entry at tissue site of infection, and the replication process in the infected cells) [11, 12]. High-resolution microscopic studies are essential in identifying the etiological agent of several outbreaks [13]. Particularly, in the case of SARS-CoV-2, the SEM images provide fundamental data of the structural aspects of the virus and must be a guiding point in therapeutic developments, for instance, in advanced antiviral drugs and monoclonal antibodies therapies [11].

The application of AFM to viruses-associated to human pathologies might have a significant impact on the diagnosis and treatment. Nowadays, scanning probe microscopy is a well-established technique for the rapid visualization of pathogens, including viruses at high resolution [14].

## Methods

This study aimed to describe the morphologic characteristics of the SARS-CoV-2 present in human nasopharyngeal specimens using high-resolution microscopy.

### Clinical specimens

A total of 12 nasopharyngeal swabs tested by Real Time-PCR to SARS CoV-2 were studied. The first six, with a negative Real-Time-PCR result, was collected from a contacts of a confirmed COVID-19 patients (with a second negative PCR result after 21 days). While the other six samples belongs to a confirmed COVID-19 patients and resulted in a positive Real -Time-PCR. These samples were received and tested at the National Reference Laboratory of Viral Respiratory Infections of the Institute of Tropical Medicine for virologic diagnostic. Real Time-PCR was performed as previously described for most common respiratory virus [15] and for SARS-CoV2 [16].

### Inactivation of clinical specimens

200 µL of the clinical specimens were inactivated for 12 h in a solution of 25% formaldehyde and 5% glutaraldehyde before microscopy study. Inactivated samples were processed at the Center for Advanced Studies of Cuba by SEM, CM, and AFM.

### Scanning electron microscopy

Ten microliters of the inactivated clinical specimen were placed in a glass-coverslip and dry-in air oven overnight. Then, the coverslips were fixed with 5% glutaraldehyde and dehydrated through a series of increasing concentrations (25–100%) of ethanol. Coverslips were further subjected to critical point drying for 1.5 h and left in a 37 °C oven overnight. Subsequently, the coverslips were sputter-coated with gold (thickness of 10 nm) and viewed under the MIRA3-TESCAN Scanning Electron Microscope (TESCAN, Czech Republic) at 10 kV.

### Atomic force microscopy

Inactivated clinical samples were processed similarly. Normally, samples for the Atomic Force Microscopy should be subjected to minimal processing to maintain its original condition. However, because of the biohazard of SARS-CoV-2, fixed, and gold-coated samples were used for this technique. The di-Innova Scanning Probe Microscope (Veeco Instruments, Santa Barbara, California) was used in tapping mode. Golden silicon probes NSG30-A, supplied by NT-MDT (Zelenograd, Russia), with a curvature radius of 10 nm and a resonant frequency of 240–440 kHz were used.

### Confocal microscopy

For the immunofluorescence staining, inactivated clinical samples were hydrated for 10 min in PBS and incubated with PBS-Tween (PBS-T) for 20 min. To block non-specific antibody reaction, the best results were obtained by incubating the sections with 0.2% bovine serum albumin (free of IgG) (Sigma Chemical Co. St. Louis, Mo. USA), for 20 min. After two washes in PBS-T, samples were incubated for 1 h at 4 °C with the primary antibody (hyper-immune serum of the COVID-19 -convalescent Cuban patient, dilutions 1:40 in PBS-T). Incubations were followed by washes with PBS-T. The second incubation was accomplished with FITC-conjugated Anti-human Polyvalent Immunoglobulins (IgA-IgG-IgM (dilutions 1:40 in PBS-T, Sigma Co. St. Louis, Mo.USA) for 1 h. After three washes with PBS-T, the sections from all samples were counterstained with propidium iodide (dilution 1:1000, Vector laboratories, Inc. Burlingame CA., USA), followed by extensive washing in PBS-T. Immunostained samples were coverslipped in Vectashield mounting medium (Vector Laboratories, Inc. Burlingame, CA., USA), Fluorescent images were observed on a Confocal laser scanning microscope OLYMPUS FV1000 IX81. Background correction of the images was performed in both, control and samples images using the Olympus Flowview FV-ASW. Software version 3.1 (Olympus, Japan).

### Improvement and segmentation of coronavirus images by mathematics algorithm [17]

In this section briefly described the mathematical algorithms used in the enhancement of microscopic images of the novel coronavirus.

The *Gaussian* filter was used to diminishing the noise in the original images. In this case, the best performance was obtained using σ = 1.5. The used window size was 3 × 3. A larger dimensional window caused a loss of information in the microphotographs (I).

#### **Definition 1**

*(Grayscale reconstruction)* The grayscale reconstruction $\rho_{I} (J)$ of *I* from *J* obtained by iterating grayscale dilations of *J* “under” *I* until stability is reached (II), that is,

1 $$\rho_{I} (J) = \mathop \vee \limits_{n \ge 1} \delta_{I}^{(n)} ( J )$$

#### **Definition 2**

The *h-dome* image *D*_*h*_(*I*) of the *h-domes* of a greyscale image ***I*** given by

2 $$D_{h} \left( I \right) \, = \, I - {\uprho }_{I} {\text{ ( I }} - {\text{h )}}$$

In expressions (1) and (2), the letters ***J ***and* I* are two grayscale images, while the symbol “*V”* means the pointwise maximum and $\delta_{I}^{\left( 1 \right)}$ is an elementary geodesic dilation.

The *h-dome* transformation extracts light structures without involving any size or shape criterion. The only parameter (*h*) related to the height of these structures. In the case of coronavirus *S-spikes* enhancement, this parameter was of very importance.

## Results

**Fig. 1:**
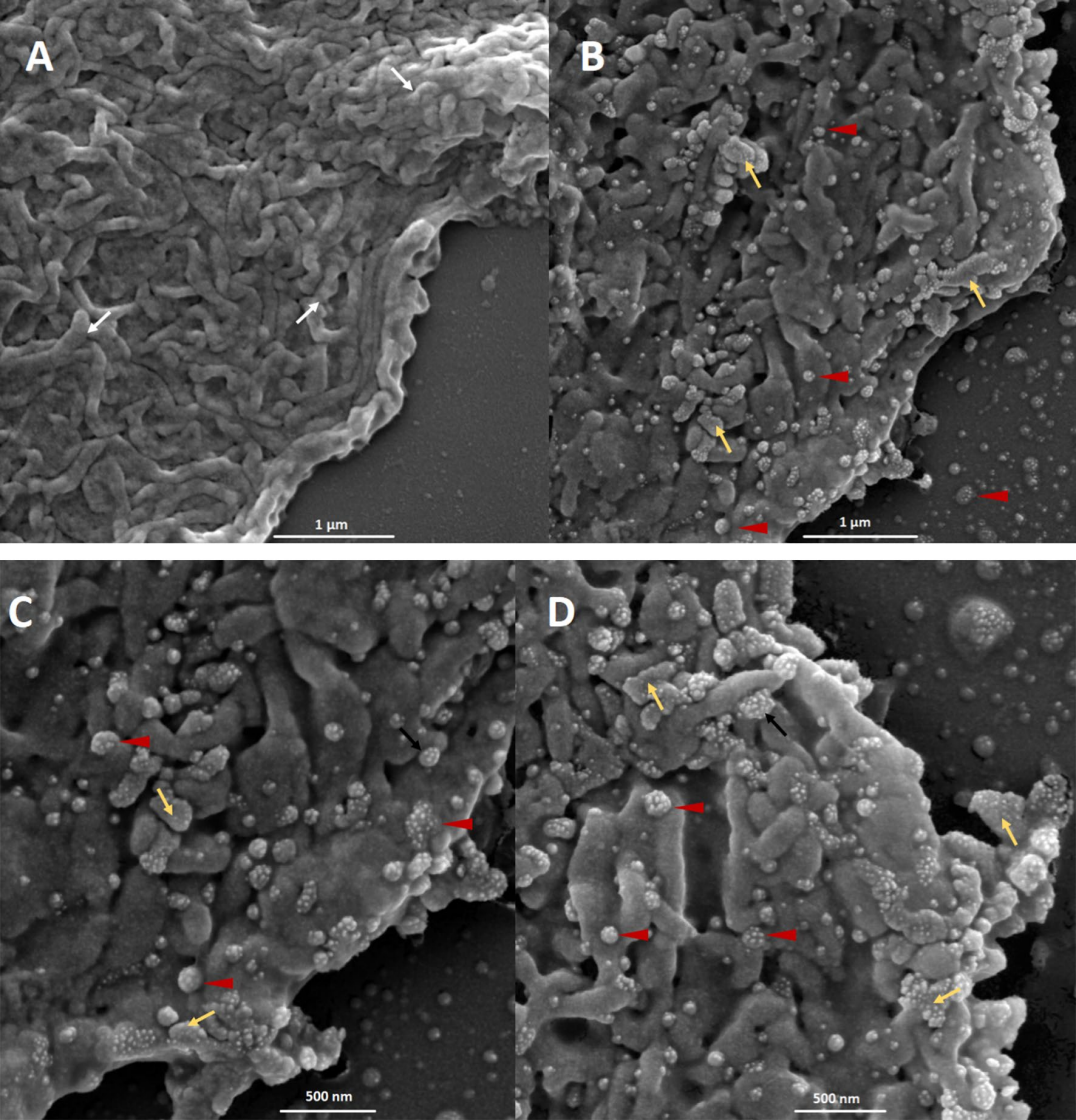
Microphotography performed with an electronic scanning microscope of the apical surface of nasal mucosal epithelial cells. **A** Sample from a negative SARS-CoV-2 individual; **B**–**D** sample from a SARS-CoV-2 positive patient. The white arrows point to the cell microvilli; red arrow heads point to the SARS-CoV-2 virus and yellow arrows point to the pseudopodia formation. Bars 1 µm–500 nm

The SEM images showed a general view of SARS-CoV-2 infected human cells. The virus-induced morphological changes demonstrated by the destruction of epithelial cells microvilli in the all positives samples (Fig. 1 and Additional files: 1–7). Uninfected cells show a typical rough surface morphology under the scanning electron microscope and no pseudopodia are visible either on the cell edge or surfaces (Fig. 1A).

**Fig. 2:**
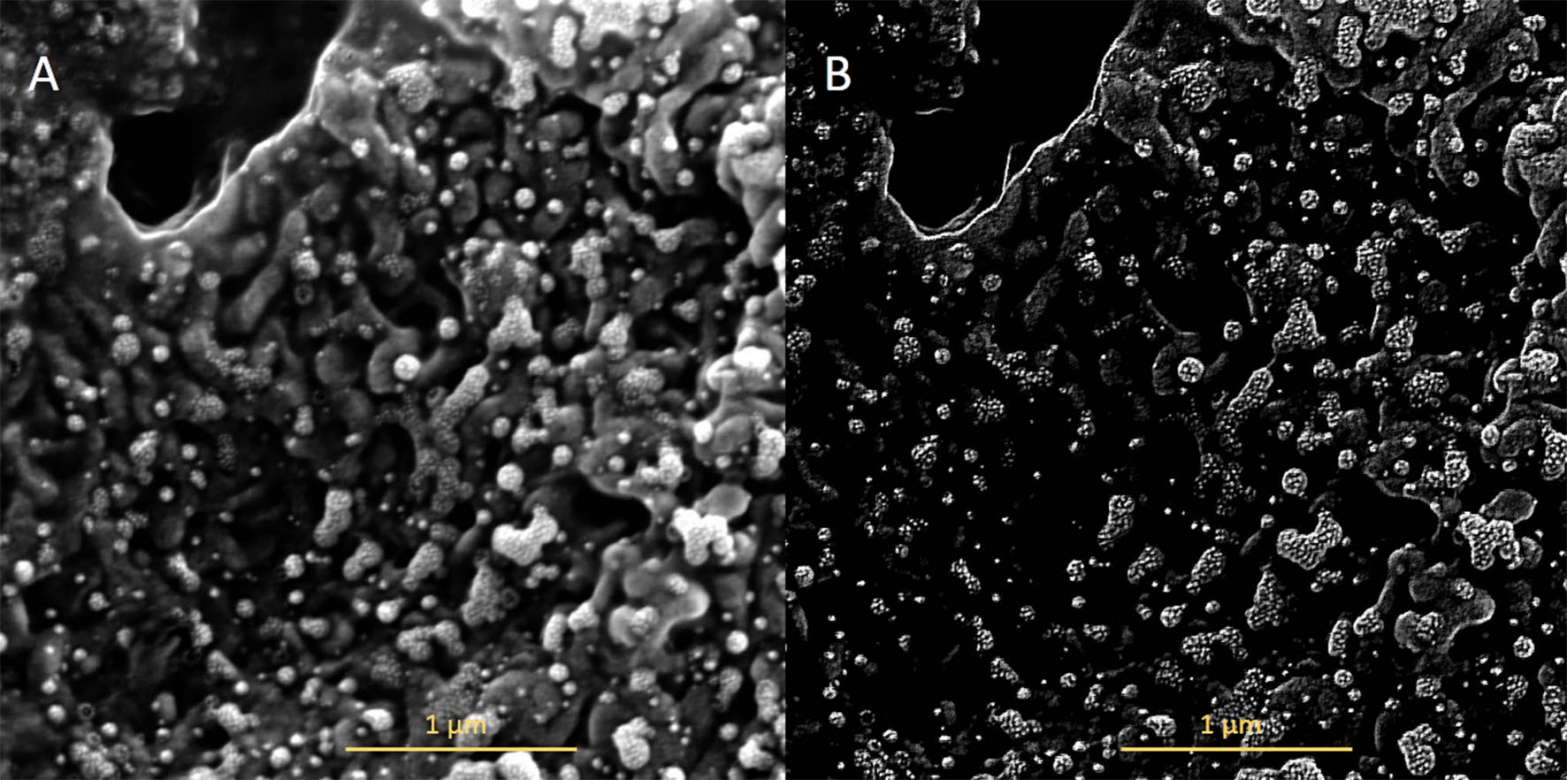
Improvement and segmentation of coronavirus images by mathematics algorithm. Microphotograph performed with an electronic scanning microscope of the apical surface of an epithelial cell of the nasal mucosa. **a** Original images; **b** improved images by mathematics algorithm where S-spikes are more visible, refining the urchin-shape in all virion particles. Bar 1 µm

The viral budding through the cell membrane was evident particularly at the edge of these cells. Different stages of the budding process were observed (Fig. 1A, B). Some areas show a high density of S-spike budding and pseudopodia projections appear to be related to an active zone of viral budding. The active budding and release of virions are more evident in the apical membrane of the epithelial cells. The whole viruses are well visualized in the lateral and basal membranes. Figure 2 shows the different stages of the viral budding process and urchin-shape of virions after the improvement and segmentation of coronavirus images by mathematic algorithms.

The viral particle size varied from 86 to 400 nm, but typical virions, observed in the cell-free space, had a size of approximately 80 nm (data no shown). At a higher magnification, crown-shape is evident in some viral particles, resembling the typical morphology of the coronaviruses (Fig. 1C, D).

No lymphocytes were observed in any of the 50 viewed-fields, suggesting a low local inflammatory immune response (data not shown).

**Fig. 3:**
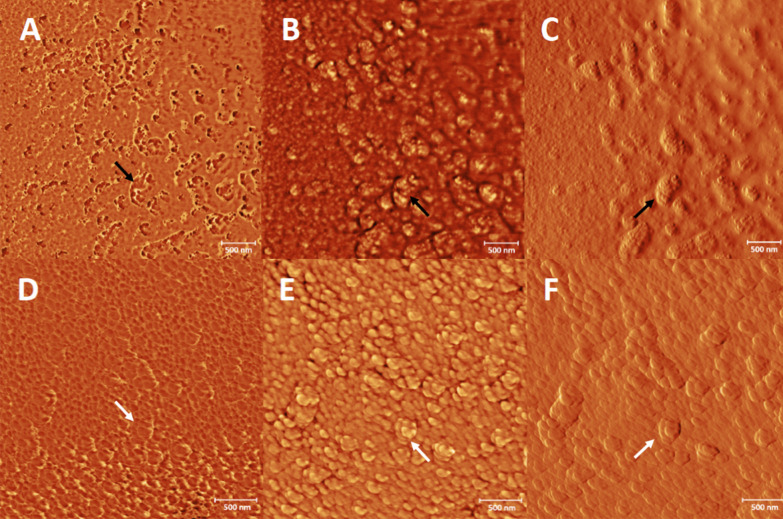
Atomic force microscopy images of an epithelial cell of the nasal mucosa of a SARS-CoV-2 infected patient. **A**, **D** phase mode images; **B**, **E** height mode images; **C**, **F** amplitude mode images. The black arrows point to SARS-CoV-2 S-spike in the cell membrane and white arrows point an isolated viral particle show the typical morphology of coronavirus by AFM. Bars 500 nm

The extrusion process of SARS-CoV 2 particles from infect cells is shown in Fig. 3. By AFM, it can be seen as a group of viruses in budding and others about to come out (arrows), which indicates the presence of different populations. This confirms the obtained results through SEM.

**Fig. 4:**
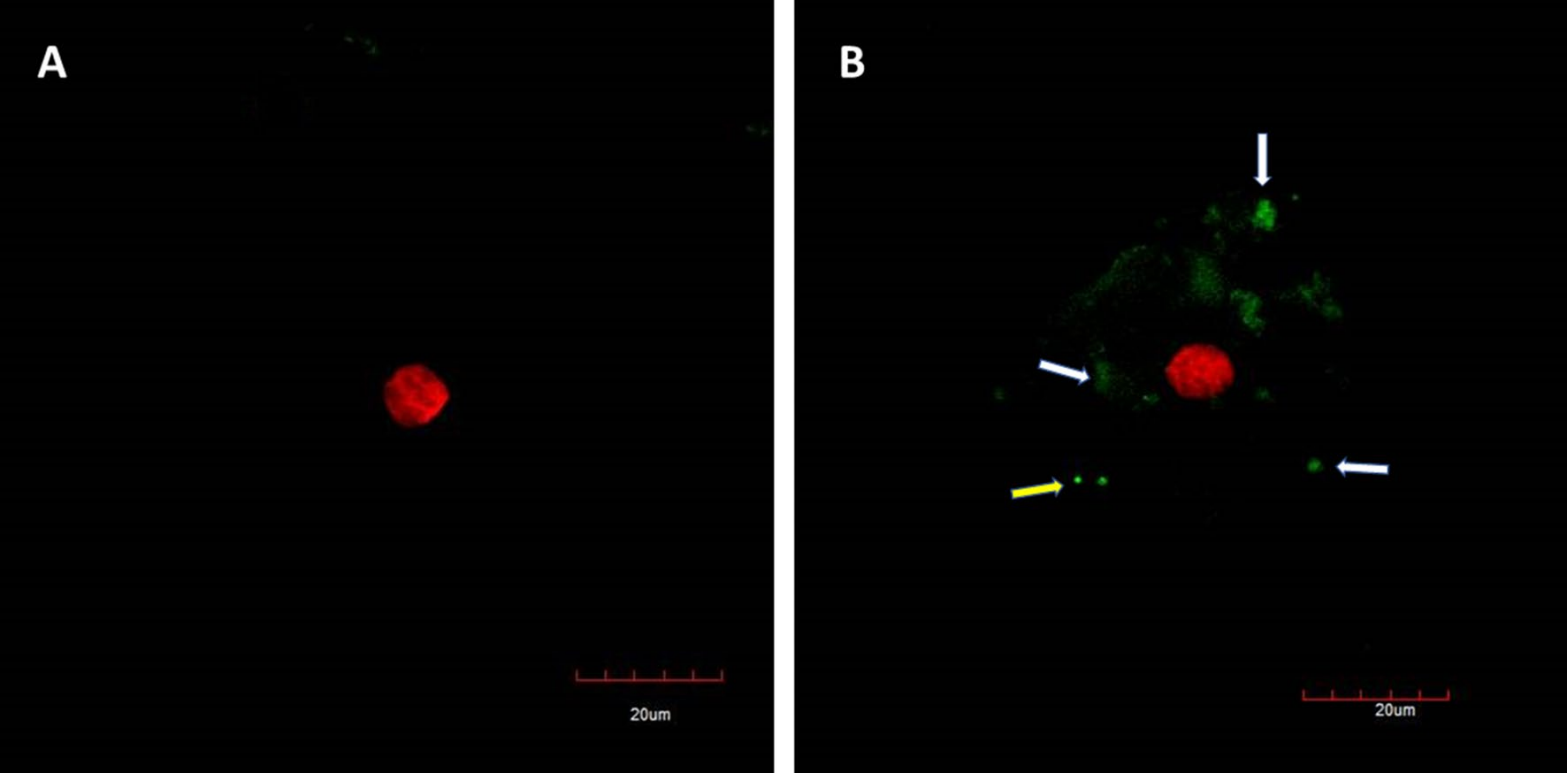
Confocal microphotograph of immunofluorescence staining of SARS-CoV-2 in epithelial cells of the nasal mucosa. **A** Sample of PCR-negative individual (negative control); **B** Sample of PCR-positive. White arrows point the virus antigen cluster coat the cell membrane and yellow arrows point the virus cluster in cell-free space. Bars 20 µm

Figure 4 shows the results of the immunofluorescence staining by confocal-microscopy. Image A represents the negative control of SARS-CoV-2. Image B, originated from the COVID-19 confirmed patient. The cells of the patient express the SARS-CoV-2 virus antigens, the antibodies present in the hyperimmune serum recognized the antigens. The positive signal of green-clouds was observed due to the presence of the viral antigens (white arrows). Clusters of antigens over the cell surface and in the free-cells space detected by CM, like to be as the virus aggrupation found by scanning electron and atomic force microscopy.

## Discussion

High-resolution SEM and AFM allow a three-dimensional holistic view of the virus and the infected cell surfaces. High-resolution microscopy is gaining popularity in other areas of the life sciences [14, 18–20]. Most of these studies were on purified macromolecules. However, the AFM has also become a virologic standard in recent years [14].

Images reconstruction provides a very efficient method to extract regional maxima and minima from grayscale SEM-images. Furthermore, the reconstruction technique extends to the determination of maximal structures, which will be call *h-domes* and *h-basins*. The h-dome transformation extracts light structure without involving any size or shape criteria. The only parameter (*h*) is related to the height of these structures. The improved images showed a wild quantity of details of virions and hypothetical cycle life stages of SARS-CoV-2 that were not seen in raw images.

The microvilli destruction observed in the infected epithelial nasal cells could be associated with the anosmia described by some patients. The active budding of the virus particles is observed mostly in the apical zone of the cells. It could be associated with the polarized translocation of immature viral particles from the Golgi apparatus as was described for SARS-CoV [6, 11, 21]. Profuse complete-virus release areas were observed in some cells (Fig. 1D). The formation of pseudopodia was recorded where the quantity of S spikes inserted in the membrane was highest with the hugest quantity of S-spike inserted in the membrane. This pseudopodia formation was reported during the multiplication of SARS-CoV in Vero cells [14].

One of the advantages of this finding is the possibility to use SEM for the follow up of long-term PCR-positive asymptomatic individuals (Mondeja, Valdes et al., unpublished results). If the virus is actively replicating in these individuals, the presence of cluster spike in the cell membrane, pseudohypha formations, and or viral particle budding, will be noted.

The non-homogeneous diameter of virus particles was observed by SEM and/or AFM, possibly due to the same unsynchronized replication of the virus. However, in the majority of the cells, virion clusters rather than isolated particles were observed. This active replication explain the high contagiosity rate of the virus that associated with an efficient mechanism of viral infection and replication leading to total cell destruction [4]. Billions of virus particles are released for a relatively long time, without much effect in the cell structure [2].

The AFM technique has shown to be a good tool for virus study. Details of the viral structure and the confirmation of the SEM finding was archived. The results of this technique in the SARS-CoV-2 study were similar to the morphological analysis of SARS-CoV previously reported [11, 14]. One explanation of the difference between SEM and AFM results of the virus-spike observation could be the previous sputtering of the sample with gold before AFM performance. For obvious reasons and the high risk of the work with non-inactivated samples carried SARS-CoV-2, the aldehyde-inactivation and gold coat were used. Fortunately, the inactivation methods do not affect the viral and cell morphology for SEM consequently this procedure can be an option to the microscopy study of clinical samples in unknow or high virulent infectious pathogens.

Confocal studies confirmed the presence of SARS-CoV-2 in clinical specimens and the distribution of the virus into the cell membrane. This is a very useful technique for virus identification using a polyclonal hyperimmune serum of convalescence patients. It could be implemented as a diagnostic assay.

It is important to note that this high-resolution microscopy study was done directly in positive SARS-CoV-2 clinical samples rather than viruses grown in cell cultures as the previous investigation of the topology of virions made in the past for SARS-CoV [11, 14]. However, our observation is very close to describing the real cellular pathology and damage of SARS-CoV-2 to the respiratory tissue in the patients. The use of hyperimmune serum of a convalescent patient could lead to the detection of other viruses, due to cross-reactivity with other co-infecting viruses such as influenza virus. Thus, it will be preferable to use a mono-specific (S protein-specific). This could be possible to cross-reactivity with another respiratory virus that could be present in the sample. If they use a different cellular receptor to ACE2 (e.g. influenza A virus), the coinfection is possible, and a false positive signal will be recorded and misunderstand as SARS-CoV-2 [8, 22–24].

Further investigations should be aimed at the nanostructure of SARS-CoV-2 by High-Resolution SEM and imaging processing to bring us a better understanding of the viral structure in clinical samples.

## Conclusions

The EM study showed that SARS-CoV-2 has a similar image to SARS-CoV. The application of several high-resolution microscopy techniques to clinical samples can help to answer important questions relating to the immunopathogenic mechanism of infection with this novel coronavirus.

## Supplementary Information

**Additional file 1: Figure S1.** Microphotography performed with an Electronic Scanning Microscope of the apical surface of nasal mucosal epithelial cells from a SARS-CoV-2 positive patient 1. Bar 500 nm.

**Additional file 2: Figure S2.** Microphotography performed with an Electronic Scanning Microscope of the apical surface of nasal mucosal epithelial cells from a SARS-CoV-2 positive patient 4. Bar 500 nm.

**Additional file 3: Figure S3.** Microphotography performed with an Electronic Scanning Microscope of the apical surface of nasal mucosal epithelial cells from a SARS-CoV-2 positive patient 5. Bar 500 nm.

**Additional file 4: Figure S4.** Microphotography performed with an Electronic Scanning Microscope of the apical surface of nasal mucosal epithelial cells from a SARS-CoV-2 positive patient 8. Bar 5 µm.

**Additional file 5: Figure S5.** Microphotography performed with an Electronic Scanning Microscope of the apical surface of nasal mucosal epithelial cells from a SARS-CoV-2 positive patient 8. Bar 1 µm.

**Additional file 6: Figure S6.** Microphotography performed with an Electronic Scanning Microscope of the apical surface of nasal mucosal epithelial cells from a SARS-CoV-2 positive patient 1. Bar 1 µm.

**Additional file 7: Figure S7.** Microphotography performed with an Electronic Scanning Microscope of the apical surface of nasal mucosal epithelial cells from a SARS-CoV-2 positive patient 11. Bar 1 µm.

## Data Availability

Not applicable.

## Abbreviations

SEM: Scanning electron microscopy
AFM: Atomic force microscopy
PBS-T: PBS-Tween-Phosphate Buffer Saline
