# Supplementary figures and images for: SARS-CoV-2: preliminary study of infected human nasopharyngeal tissue by high resolution microscopy
Source: Virol J. 2021 Jul 18;18:149. doi: 10.1186/s12985-021-01620-1 (PMC8286443; doi:10.1186/s12985-021-01620-1)

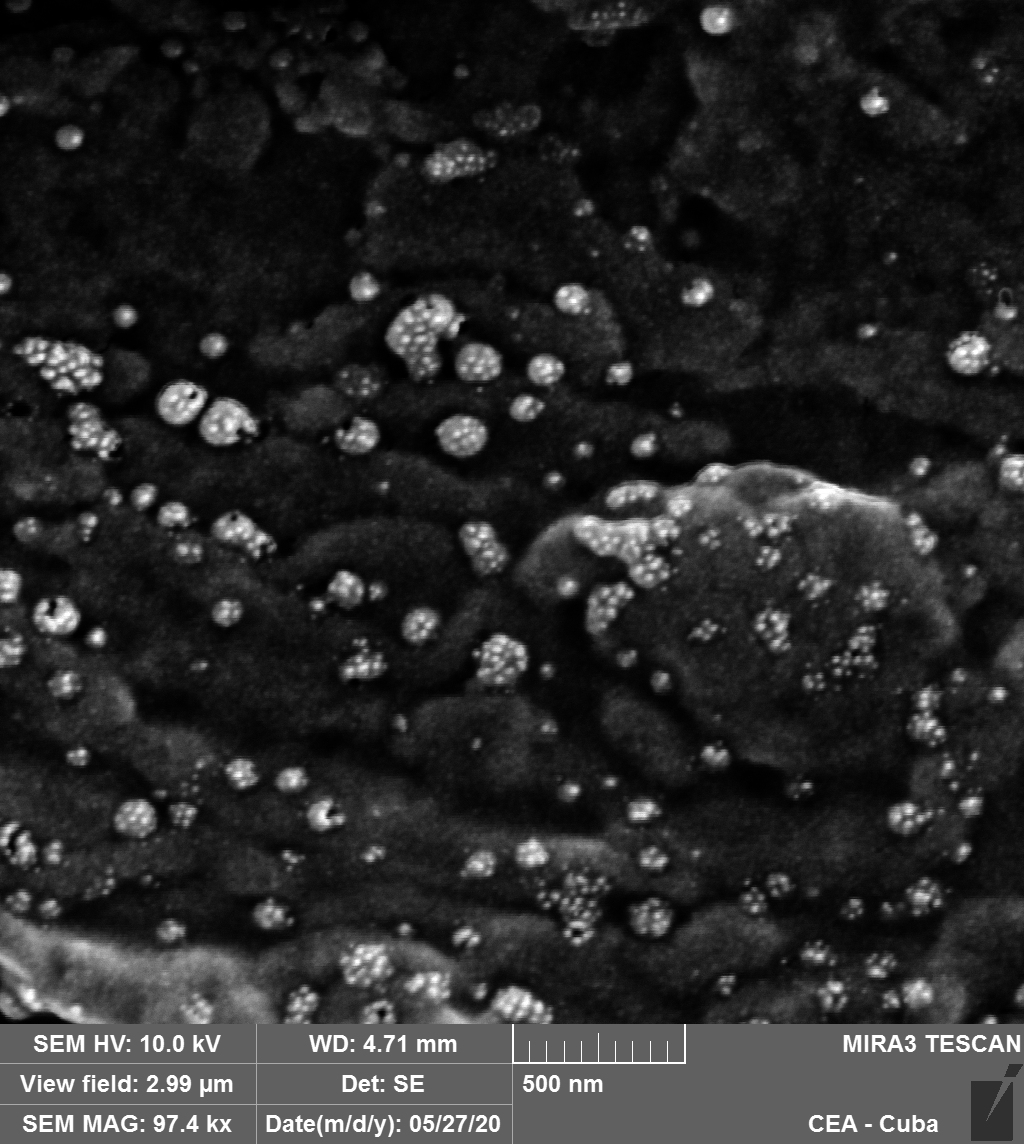

Supplement: Supplementary file 1 — Additional file 1: Figure S1. Microphotography performed with an Electronic Scanning Microscope of the apical surface of nasal mucosal epithelial cells from a SARS-CoV-2 positive patient 1. Bar 500 nm. [file 12985_2021_1620_MOESM1_ESM.tif]

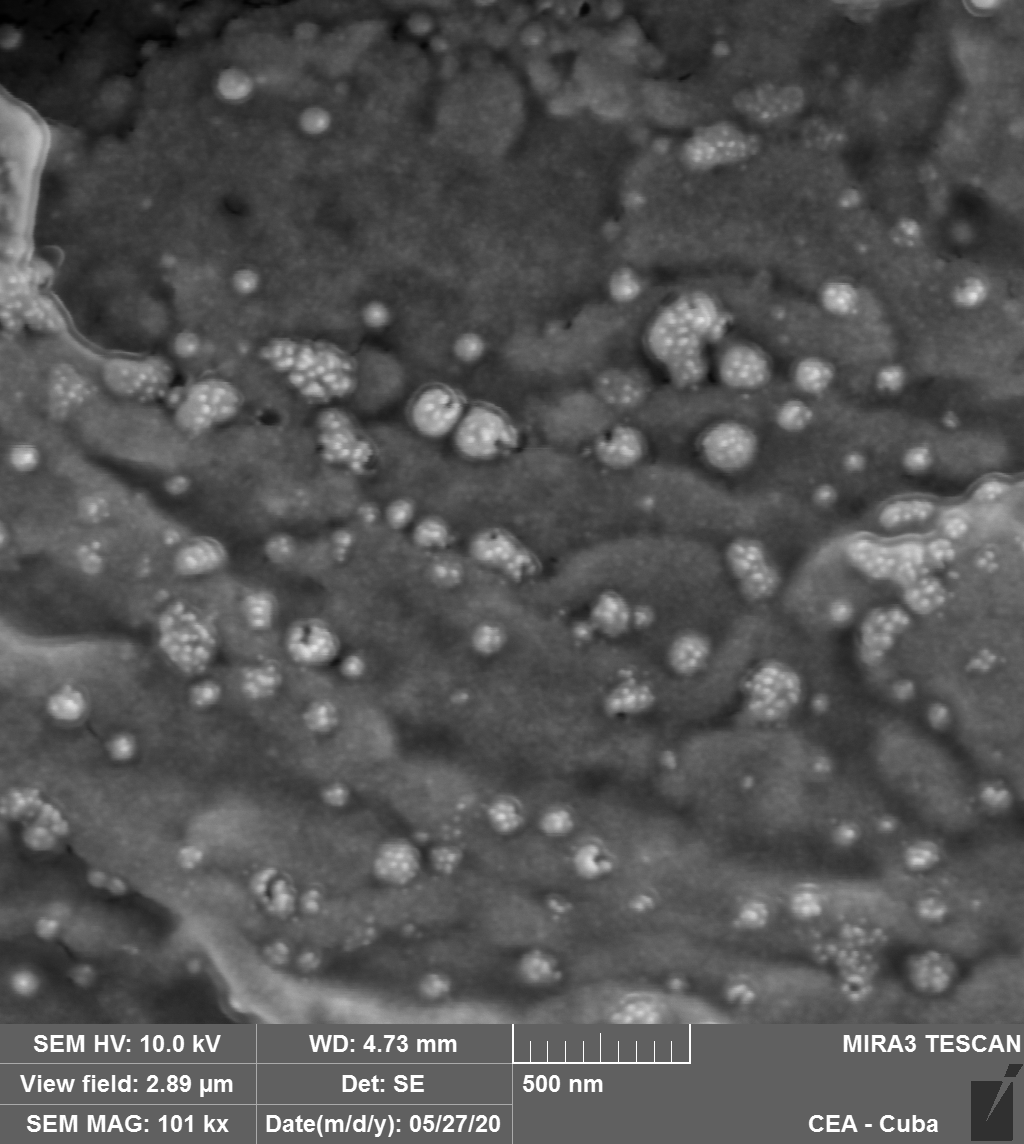

Supplement: Supplementary file 2 — Additional file 2: Figure S2. Microphotography performed with an Electronic Scanning Microscope of the apical surface of nasal mucosal epithelial cells from a SARS-CoV-2 positive patient 4. Bar 500 nm. [file 12985_2021_1620_MOESM2_ESM.tif]

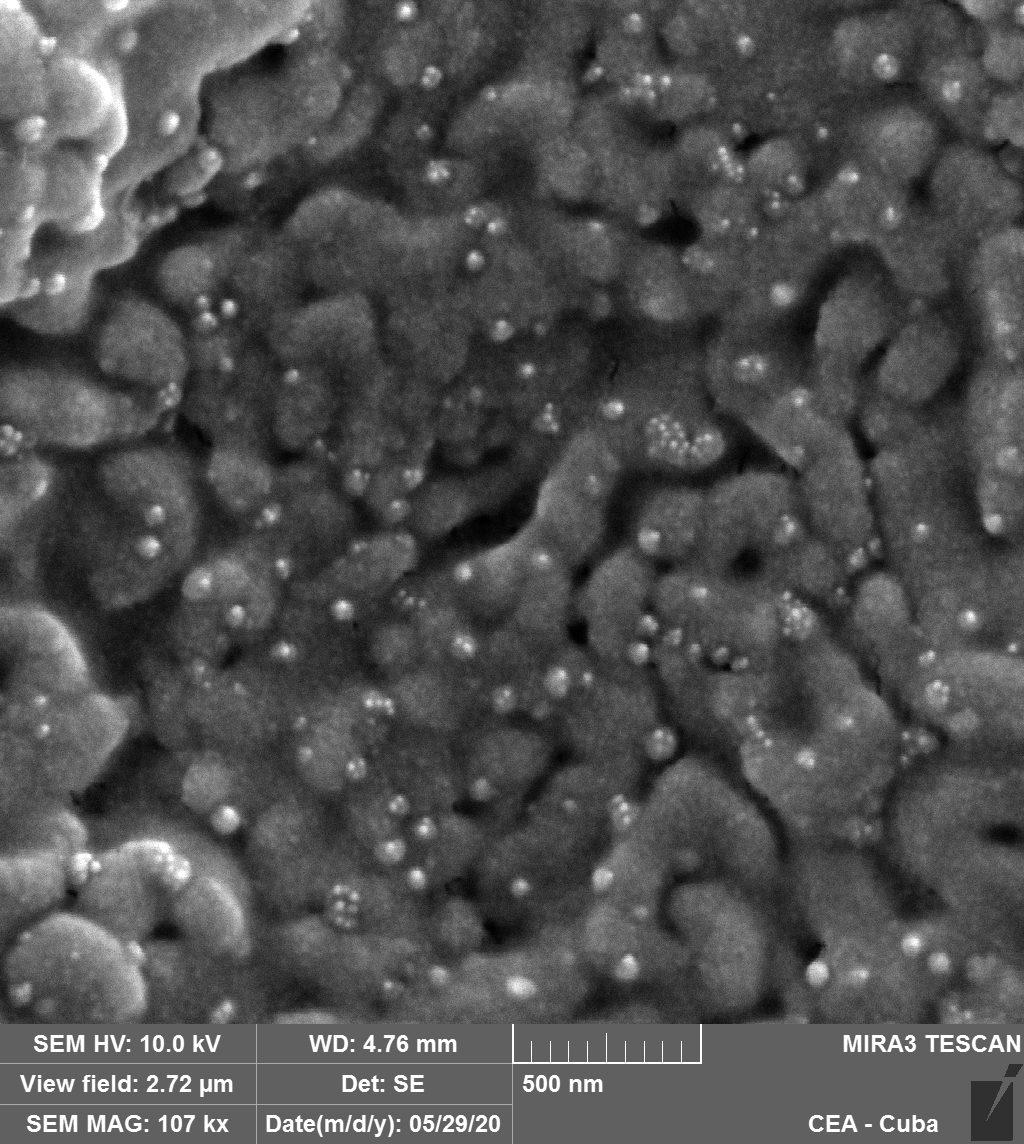

Supplement: Supplementary file 3 — Additional file 3: Figure S3. Microphotography performed with an Electronic Scanning Microscope of the apical surface of nasal mucosal epithelial cells from a SARS-CoV-2 positive patient 5. Bar 500 nm. [file 12985_2021_1620_MOESM3_ESM.tif]

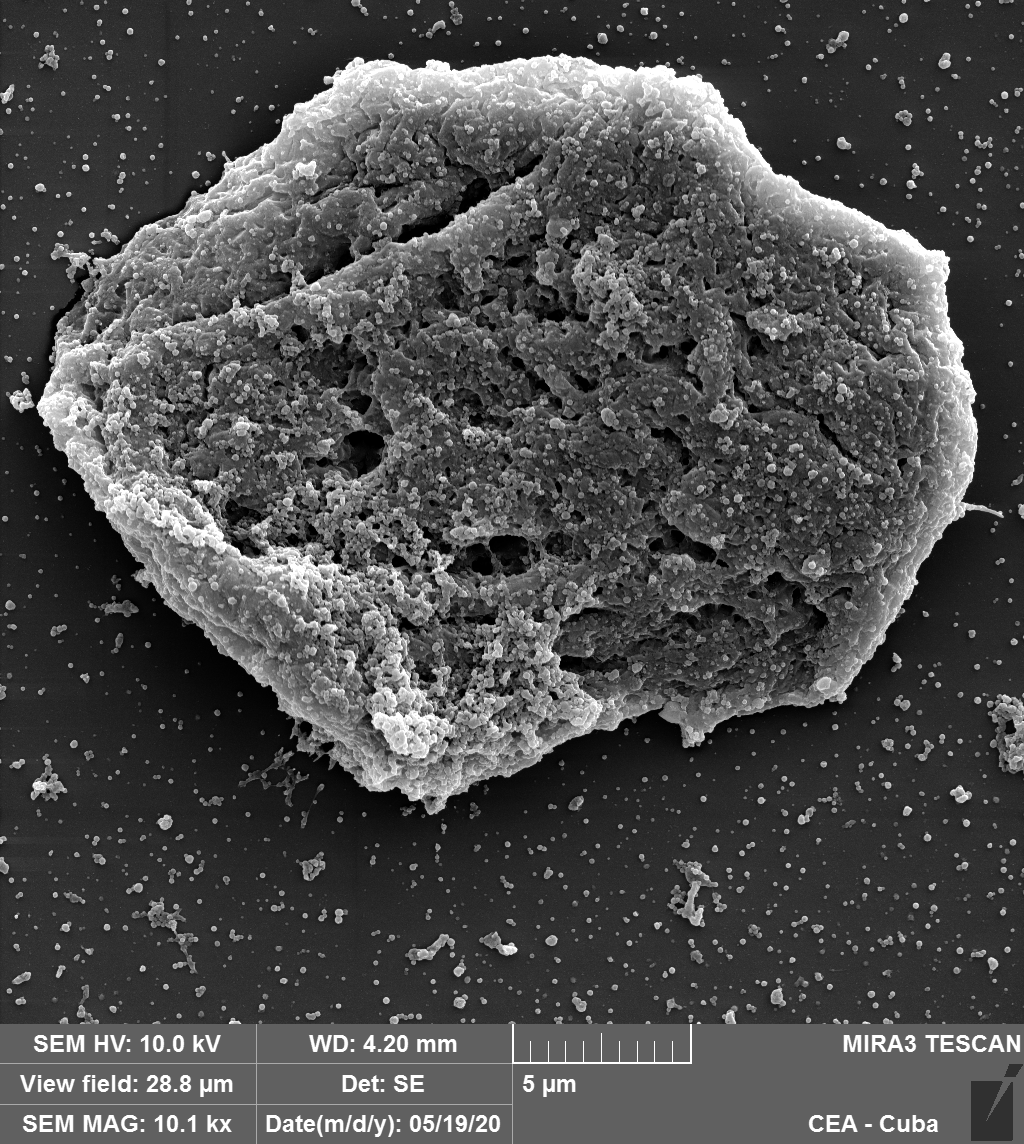

Supplement: Supplementary file 4 — Additional file 4: Figure S4. Microphotography performed with an Electronic Scanning Microscope of the apical surface of nasal mucosal epithelial cells from a SARS-CoV-2 positive patient 8. Bar 5 µm. [file 12985_2021_1620_MOESM4_ESM.tif]

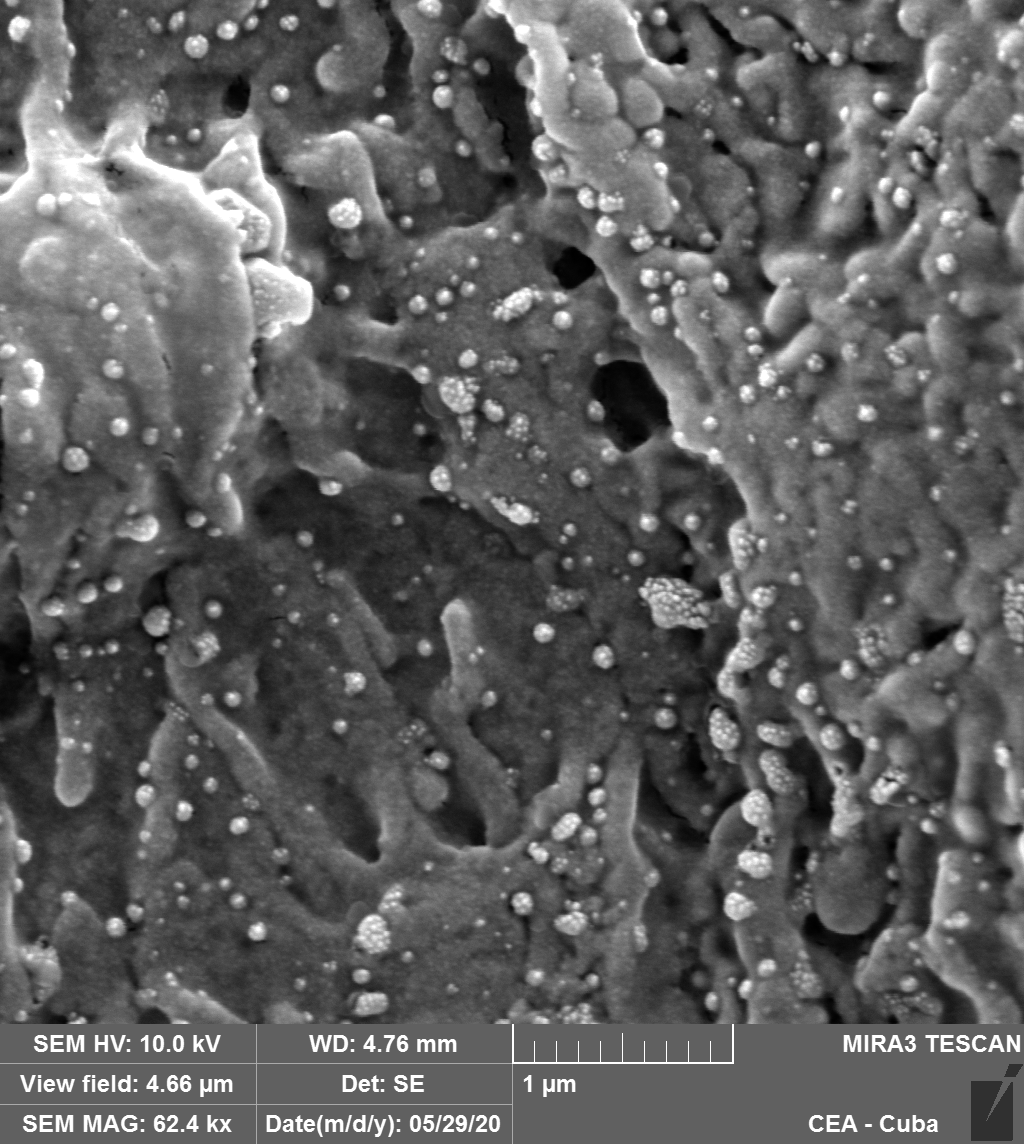

Supplement: Supplementary file 5 — Additional file 5: Figure S5. Microphotography performed with an Electronic Scanning Microscope of the apical surface of nasal mucosal epithelial cells from a SARS-CoV-2 positive patient 8. Bar 1 µm. [file 12985_2021_1620_MOESM5_ESM.tif]

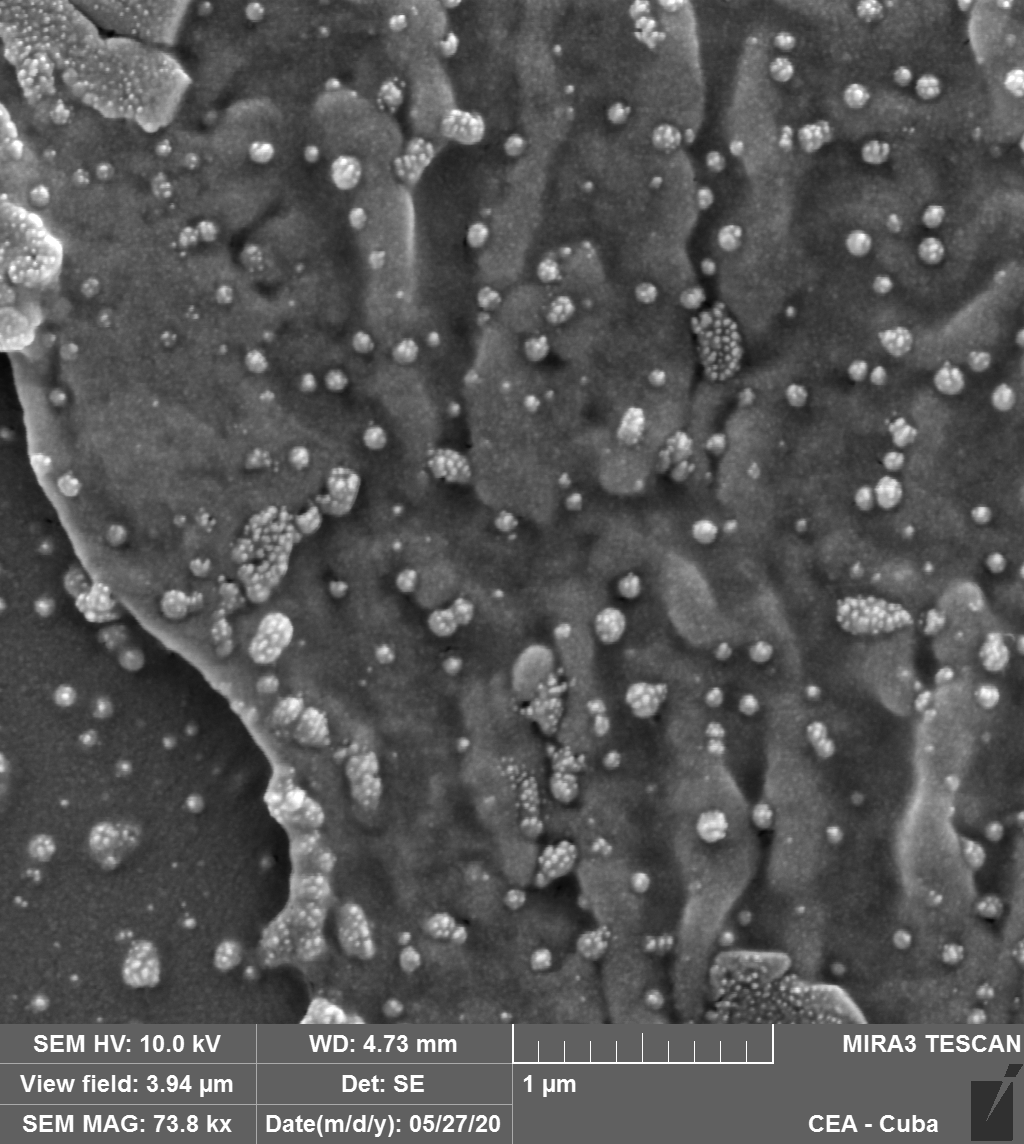

Supplement: Supplementary file 6 — Additional file 6: Figure S6. Microphotography performed with an Electronic Scanning Microscope of the apical surface of nasal mucosal epithelial cells from a SARS-CoV-2 positive patient 1. Bar 1 µm. [file 12985_2021_1620_MOESM6_ESM.tif]

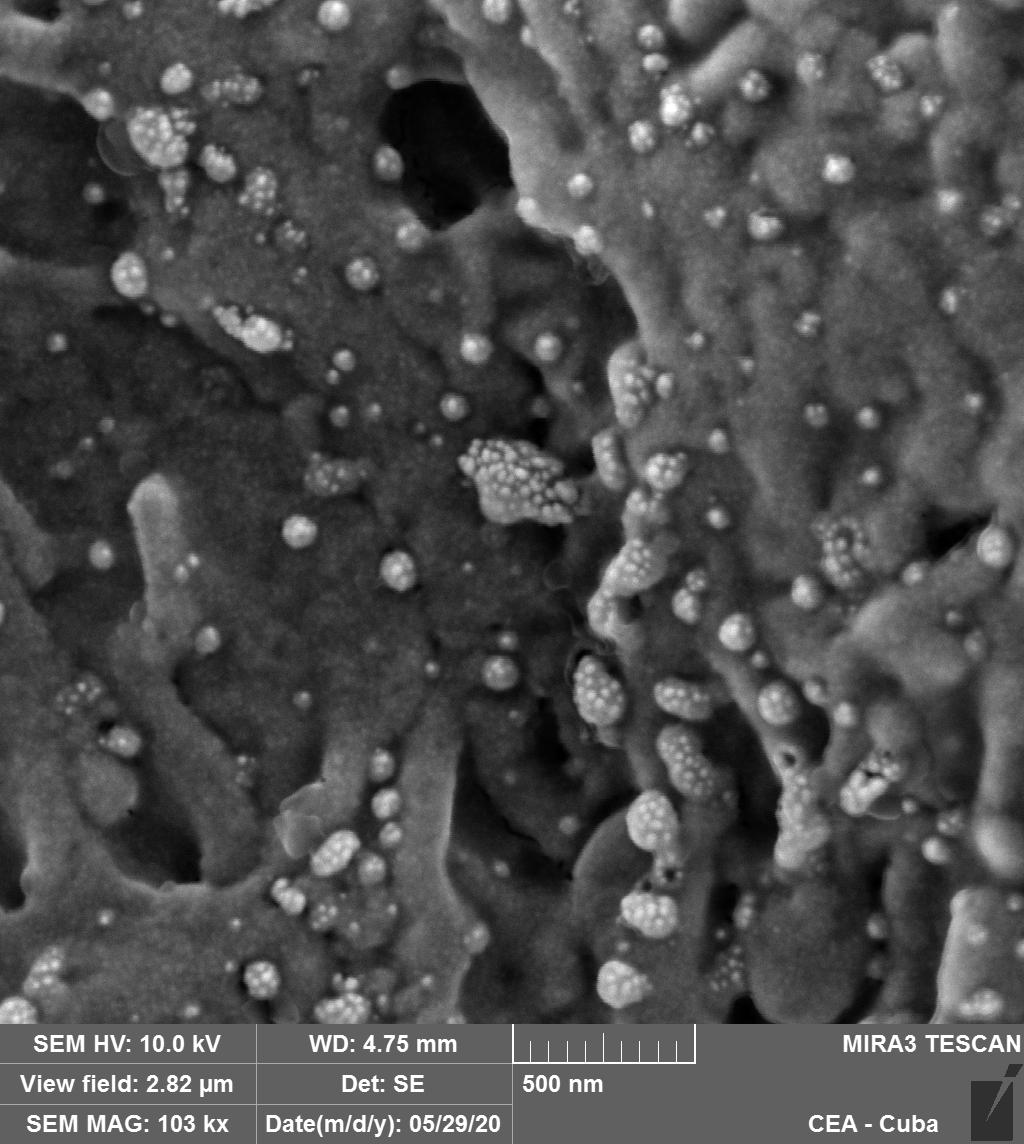

Supplement: Supplementary file 7 — Additional file 7: Figure S7. Microphotography performed with an Electronic Scanning Microscope of the apical surface of nasal mucosal epithelial cells from a SARS-CoV-2 positive patient 11. Bar 1 µm. [file 12985_2021_1620_MOESM7_ESM.tif]
